# Evaluation of the BALANCE Program as a Digital Therapeutic Solution for Type 2 Diabetes Management: Protocol for a Prospective Lifestyle Intervention Study

**DOI:** 10.2196/73964

**Published:** 2025-12-08

**Authors:** Jane Tey, Joshua Lam, Chee Kwang Yung, Musjarena Abd Mulok, Nurshazwani Mat Salleh, Alice Moi Ling Yong, Yvonne Lee, Pui Lin Chong

**Affiliations:** 1 EVYD Technology Sdn Bhd Bandar Seri Begawan Brunei Darussalam; 2 Endocrine Centre Raja Isteri Pengiran Anak Saleha Hospital Ministry of Health Bandar Seri Begawan Brunei Darussalam; 3 Department of Health Services Ministry of Health Bandar Seri Begawan Brunei Darussalam

**Keywords:** BALANCE program, type 2 diabetes mellitus, digital intervention, digital therapeutics, mobile health, mHealth, diabetes mellitus digital therapeutics, DM DTx, health coaching, chronic disease management

## Abstract

**Background:**

Type 2 diabetes mellitus (T2DM) is a growing global health concern. In 2016, 9.7% of Bruneian adults aged 18 to 69 years had diabetes, making it the third leading cause of death. Effective self-management can mitigate complications that require health care interventions and lower health care costs. Brunei Darussalam has deployed BruHealth, a national mobile health platform synced with the Brunei Health Information Management System, allowing patients to access health records, schedule appointments, and explore medical articles. A digital therapeutics module for T2DM (diabetes mellitus digital therapeutics [DM DTx]) has been developed, consisting of a digital lifestyle intervention module within BruHealth and a separate health care professional portal for health coaches. The 16-week BALANCE program aims to support self-management. This study explores the efficacy of DM DTx in managing T2DM through digital lifestyle interventions.

**Objective:**

The primary objective is to determine the proportion of participants who achieve at least a 0.6% reduction in glycated hemoglobin after 16 weeks. Secondary objectives include evaluating changes in glycated hemoglobin, fasting lipid profile, blood glucose, BMI, and waist circumference and analyzing participant feedback. Given the predominantly Muslim population, the study also aims to gain insight into fasting practices for Muslim participants with T2DM.

**Methods:**

This single-arm, nonrandomized intervention study involves adults aged 18 to 70 years with T2DM. Participants complete a fully online 16-week program via BruHealth that includes diabetes self-management education, personalized diet and exercise plans, self-monitoring tools (eg, glucometer and smartwatch), and support from health coaches through video consultations and instant messaging. Anthropometric and biochemical measures are collected at baseline and after the intervention. Data sources include the Brunei Health Information Management System, BruHealth app logs, and the health care professional portal. Descriptive statistics will summarize participant characteristics and outcomes. Paired 2-tailed *t* tests or Wilcoxon signed-rank tests will compare the results before and after the intervention. Subgroup analyses will explore outcomes based on glycemic changes, BMI, medication type, and program engagement. Participant feedback will be qualitatively analyzed. Fasting risk in Muslim participants will be stratified using the International Diabetes Federation-Diabetes and Ramadan Alliance Risk score.

**Results:**

Recruitment began on August 20, 2024, following project approval in July 2024, and continued until July 2025. By the end of data collection on November 12, 2025, a total of 459 participants had been enrolled, and 422 (91.9%) had completed the program. Data analysis is currently ongoing, with results expected in early 2026.

**Conclusions:**

Self-management mobile health apps are promising tools for chronic disease management, including T2DM. The BALANCE program is Brunei’s first national-scale study evaluating a fully online T2DM intervention. Localization to a region’s population may support improved health outcomes.

**International Registered Report Identifier (IRRID):**

DERR1-10.2196/73964

## Introduction

### Background

Type 2 diabetes mellitus (T2DM) has emerged as a significant global health concern, with a worldwide prevalence of 537 million adults in 2021, and it is projected to rise to 783 million by 2045 [1]. In 2016, 9.7% of Bruneians aged between 18 and 69 years were diagnosed with diabetes [2]. Diabetes is the third leading cause of death in Brunei Darussalam, accounting for 10.1% of total deaths in 2017 [3]. Therefore, there is a need for effective management strategies tailored for patients with T2DM to achieve target glycemic control and reduce the risks of developing complications. This will lead to more efficient use of health resources.

In Brunei Darussalam, diabetes-related health care costs strain the national budget and heighten demand for services necessary to manage complications related to diabetes. The global cost of diabetes was estimated at US $966 billion in 2021 and is projected to rise to US $1.03 trillion by 2045 [1]. This burden includes direct medical expenses and indirect costs from increased prescription use, costly hospital services, and complications that can reduce overall productivity and lead to unemployment [4,5].

T2DM is a chronic disease that can lead to severe complications if not managed appropriately [6]. This underscores the critical role of self-management. Effective lifestyle management, which incorporates regular physical activity, a healthy diet, and medication adherence, can reduce both disease complication risks and health care costs [7]. By using SMART (specific, measurable, achievable, relevant, and time-bound) goals and behavioral techniques, individuals can improve their diabetes control and disease outcomes [8]. Recent studies have demonstrated that digital health interventions favorably impact glycemic control, patient adherence, and engagement [9,10]. Additionally, mobile health (mHealth) apps have been shown to positively improve health self-management practices on chronic conditions [11-13].

The digitalization of health care can further benefit the management of chronic diseases, such as T2DM [14]. With mobile ownership reaching 78% globally [15], health care can access a broad audience, enhancing health equity by providing vital information and resources. This accessibility equips underserved populations with tools for effective self-management, such as tracking health metrics and connecting with health care providers for personalized guidance.

BruHealth is the national mHealth app and health care portal in Brunei Darussalam, initially developed to mitigate COVID-19 spread. It has since evolved into a comprehensive digital health solution, benefiting the general population. The app is synchronized with the Brunei Darussalam Health Care Information and Management System (BruHIMS), where 95% of the population has active health records, allowing real-time access to health care data. Users can view their health records through BruHealth, enabling them to monitor their health status and access their blood test results. Beyond pandemic-related features, BruHealth now offers online appointment scheduling, health assessments, and access to medical articles on topics such as nutrition and lifestyle. By providing an accessible, user-friendly platform, BruHealth promotes the adoption of digital health technologies while allowing users to manage their health effectively [16].

Most existing global digital therapeutics (DTx) apps function as stand-alone tools, often relying on self-reported data and offering limited integration with national health systems. These apps frequently incur additional costs to users and typically focus on a single behavioral domain, such as diet tracking or medication adherence, thereby limiting their impact. The BALANCE program, delivered through BruHealth, adopts a holistic and culturally contextualized approach to diabetes prevention and management. The program addresses multiple lifestyle domains and provides education that has been adapted to the lifestyle in Brunei. Through integration with reliable wearables and BruHIMS, data captured can be used to create an appropriate health plan, enabling users to monitor their condition more seamlessly. This centralized and personalized system overcomes the key limitations of conventional DTx tools, particularly by offering a tailored and accessible solution for Brunei’s population [17]. The digitalization of this tool also increases accessibility to services and education that would otherwise be available only in person.

### Rationale

DTx are software-based treatments that deliver interventions with the best available evidence to manage or prevent diseases. They supplement or replace traditional therapies and are prescribed for chronic conditions such as diabetes and mental health disorders. In Brunei Darussalam, a DTx module focused on the management of T2DM (diabetes mellitus DTx [DM DTx]) has been developed.

The benefits of the DM DTx include patient education on diabetes management through lifestyle adjustments for better glycemic control, and real-time advice that has been designed to seamlessly integrate into daily activities, thus enabling patients to self-manage their diabetes with greater confidence. DM DTx may improve self-management and the quality of life of individuals with T2DM while paving the way for a more efficient and effective health care model.

The DM DTx module consists of a digital lifestyle intervention (named the BALANCE program, which is integrated into the BruHealth app) and a health care professional (HCP) portal where health coaches can interact with their patients and help them manage their condition. The BALANCE program has evolved from a previous pilot study conducted to evaluate the efficacy and feasibility of a digital lifestyle intervention for T2DM [18]. The pilot intervention program was designed with the expertise of endocrinologists and dietitians based on national clinical guidelines and evidence-based practice in diabetes management. Following further refinement and complete digitalization of the initial program, our study aims to assess the potential impact of a T2DM digital intervention that uses a fully online management approach.

Separately, as Brunei Darussalam has a Muslim-majority community, there is an optional component within the DM DTx offered to Muslim participants as an opportunistic way of gaining more insights into their fasting practices during the last Ramadan. Studies have shown that a significant number of Muslim patients in Brunei Darussalam with diabetes fast during Ramadan. For instance, a study found that 93.4% of participants fasted during Ramadan, with an average of 24.1 days. This optional component in the DM DTx could provide valuable insights into how fasting impacts diabetes management and help tailor more effective treatment plans for Muslim patients [19].

### Aims and Objectives

Our study aims to evaluate the efficacy of the DM DTx in managing patients with T2DM by delivering digital lifestyle interventions through an easily accessible platform (BruHealth), and to evaluate participants’ feedback to improve the app’s content and structure for better user experience.

The primary objective is to determine the proportion of participants who achieve at least a 0.6% reduction in glycated hemoglobin (HbA_1c_) from baseline following a 16-week lifestyle intervention provided by the DM DTx module. Secondary objectives are (1) to evaluate overall changes in HbA_1c_, fasting lipid profile, fasting blood glucose, BMI, and waist circumference over a 16-week period; (2) to analyze participants’ feedback on the BALANCE program; and (3) to evaluate fasting experience among Muslims with T2DM during Ramadan and to calculate their fasting risk using the International Diabetes Federation-Diabetes and Ramadan Alliance Risk (IDF-DAR) score [20].

## Methods

### Study Design

This is a single-arm, nonrandomized intervention study that involves the use of BALANCE, a 16-week diabetes lifestyle management program (described in more detail subsequently under the “Intervention” section). Patients with T2DM are enrolled and taught diabetes self-management with structured educational courses, and their progress is monitored through the DM DTx with dedicated health coaches.

Anthropometric measurements are taken at baseline and at the end of the study. These include BMI (height and weight) and waist circumference. A fasting blood sample is also taken at baseline and at the end of the study. The blood tests include the following:

- Fasting blood glucose
- HbA_1c_
- Fasting lipid profile (total cholesterol, triglycerides, low-density lipoprotein cholesterol, and high-density lipoprotein cholesterol)

Serum creatinine is also measured at baseline if there is no result available within 1 year of enrollment.

### Participants

#### Sample Size

By using a paired 2-tailed *t* test, a sample of 439 participants will provide 90% power to detect a 0.14 effect size at a .05 significance level. Considering a potential attrition rate of 12% based on the previous pilot study, the target sample size has been set at 500 participants.

#### Inclusion Criteria

Individuals with T2DM, aged between 18 and 70 years, either on diet control, treatment with oral hypoglycemic agents, once or twice daily insulin injections, or at least 6 months of glucagon-like peptide-1 receptor agonists are included in the study.

#### Exclusion Criteria

The following individuals are excluded from the study: (1) those with T2DM requiring more than twice daily insulin injections; (2) those with type 1 diabetes; (3) those who are pregnant or breastfeeding; (4) individuals with a recent history of recent myocardial infarction or cerebrovascular accident in the past 6 months; (5) those with a history of heart failure, liver failure, or active cancer diagnosis; (6) those undergoing treatment for active foot disease or active diabetes-related eye disease; (7) those with chronic kidney disease stage 4 or 5 according to estimated glomerular filtration rate (based on chronic kidney disease-epidemiology collaboration ); (8) those with a history of hospitalization in the past 6 months; (9) those with physician’s advice not to participate in intense physical activity; (10) those with physical disability or who are unable to perform activities of daily living; and (11) those unable to use the BruHealth app or watch YouTube videos independently on their mobile devices.

#### Recruitment

This study uses a volunteer sampling strategy. Recruitment is undertaken through the BruHealth app, which can be accessed by residents with a mobile device in Brunei Darussalam. Upon completion of the screening questionnaire on BruHealth, eligible participants will be enrolled in the study.

Online promotions comprise several methods, such as digital banners on the BruHealth app, targeted nudges to BruHealth users diagnosed with T2DM, and digital marketing strategies on social media platforms. Offline strategies include health promotional events, radio promotions, and the distribution of printed promotional materials in hospitals and health centers.

### Intervention

#### Overview

Participants who have been enrolled in the study will undergo a briefing session on the BALANCE program. During the briefing session, Muslim participants who had fasted during Ramadan in 2024 and who intend to fast this year will be assessed using the IDF-DAR risk stratification tool to determine their risk score and their safety of fasting. Risk stratification is an important step in the process of providing guidance to individuals with diabetes who fast during Ramadan. Each participant is assigned a health coach for personalized care and provided with tailored lifestyle modification recommendations on the BALANCE program.

Participants are provided with the following equipment: a glucometer, lancing device, blood test strips, and lancets to measure blood glucose; a resistance band for exercise; a measuring tape to measure waist circumference; a Bluetooth-enabled weighing scale to measure weight; and a smart watch to track step count and aerobic exercise. The glucometer, weighing scale, and smart watch are synchronized to the app to enable automatic data logging.

#### Diabetes Self-Management Education

The structured diabetes self-management education course focuses on sharing health care knowledge and tips specifically for individuals with diabetes. The aim is to increase health literacy to enable participants to be more aware of how their lifestyle choices may affect their glycemic control.

The materials are presented in the form of videos and educational articles, split across 4 phases (Textbox 1) to build upon knowledge acquired in earlier phases. There are also practice and review components to test the participants’ understanding of the materials.

**The 4 phases of diabetes self-management education across the 16-week intensive education program.**

**Phase 1 (weeks 1 to 2)**

- Pathophysiology
- Diabetes self-management
- Goal setting, motivation, and coping skills
- Hypoglycemia and hyperglycemia management, healthy eating patterns, exercising with diabetes, and precautions

**Phase 2 (weeks 3 to 6)**

- Advance nutrition education on grocery shopping, tips for special occasions, etc.
- Monitoring skills and importance and medications
- Best exercise practices and planning tips

**Phase 3 (weeks 7 to 10)**

- Diabetes-related complications and prevention
- Incorporating nutrition into daily life and eating habits
- Problem-solving skills and tips to encourage diabetes self-management

**Phase 4 (weeks 11 to 16)**

- No new courses, focus on converting learning to behavior
- Recommend articles or animations targeting barriers

#### Lifestyle Modification Plans, Logging, and Reports

##### Overview

The BALANCE program administers lifestyle modification intervention in blood glucose monitoring, exercise, and diet through personalized recommendations and goals based on participants’ current state of health and lifestyle habits. SMART goals, which are specific, measurable, achievable, relevant, and time-bound, are incorporated into the program through the design of logging functions and further reinforced through educational courses and active monitoring by the health coaches. Health coaches encourage participants to achieve lifestyle changes by ensuring that the set goals are clear, measurable, and time sensitive, which enhances motivation and accountability. This approach helps participants to clearly define and track their progress toward managing their condition more effectively through goal-setting and behavioral activation.

At the start of the program, personalized plans are generated for each participant, as described subsequently.

##### Blood Glucose Monitoring

**Figure 1:**
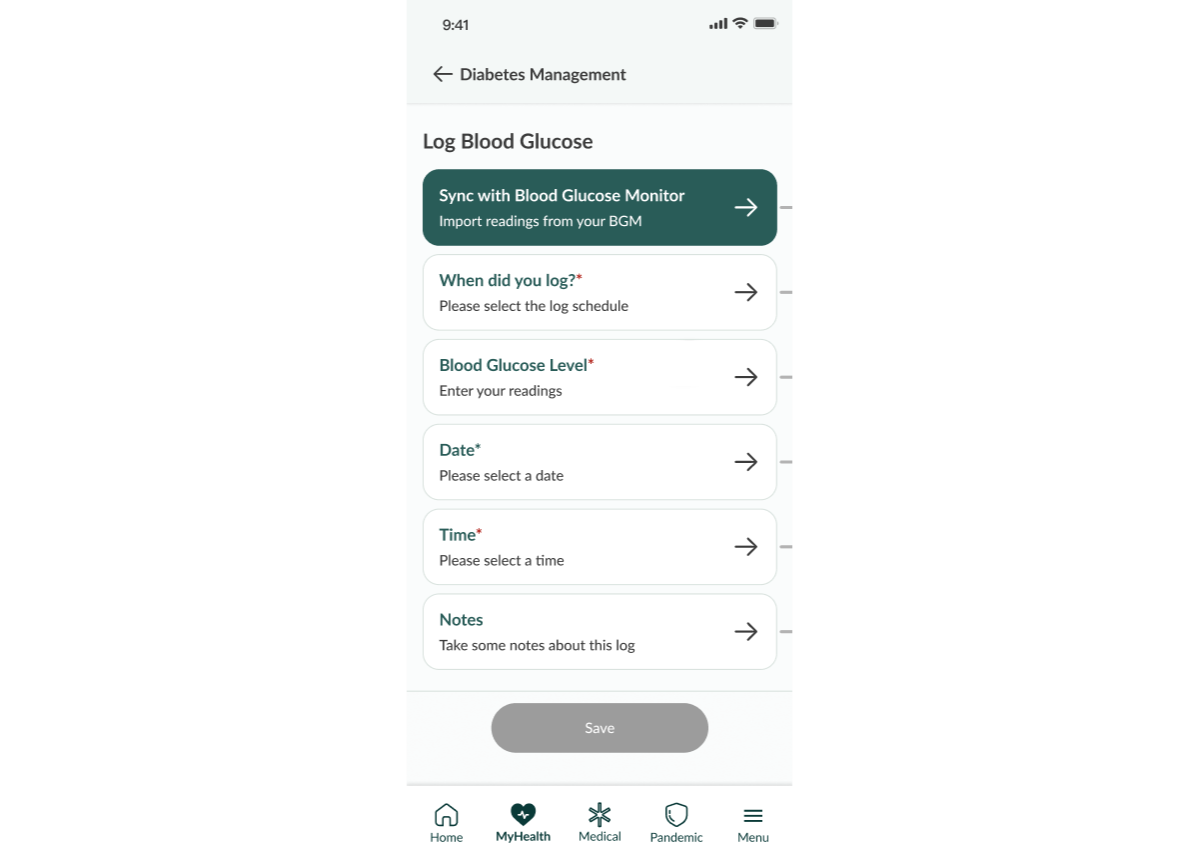
Blood glucose logging feature.

**Figure 2:**
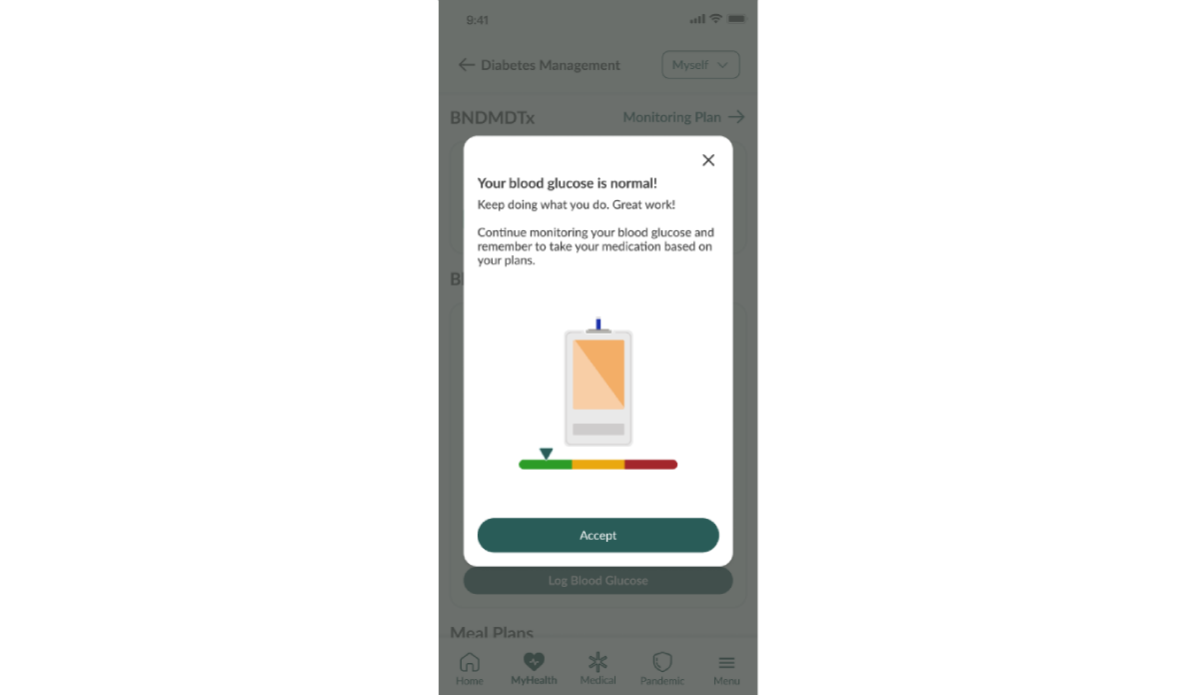
Instant feedback with blood glucose logging.

Participants are taught to undergo structured capillary blood glucose monitoring with logging of their blood glucose readings (Figures 1 and 2). Management of hypoglycemia and significant hyperglycemia (defined by our study protocol) will be discussed in the Escalation Model section. For nonsevere hypoglycemia or hyperglycemia, participants will receive self-management guidance through the DM DTx.

##### Exercise

**Figure 3:**
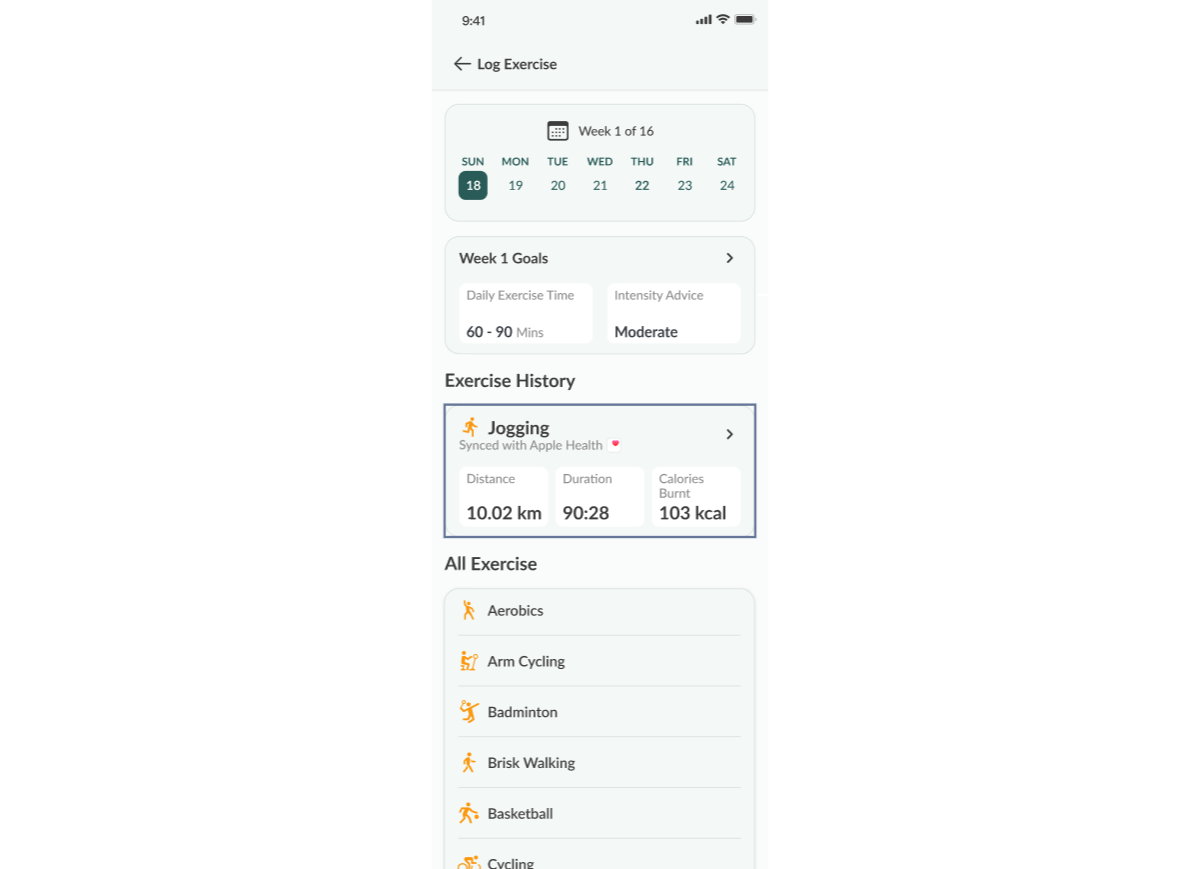
Exercise plan.

**Figure 4:**
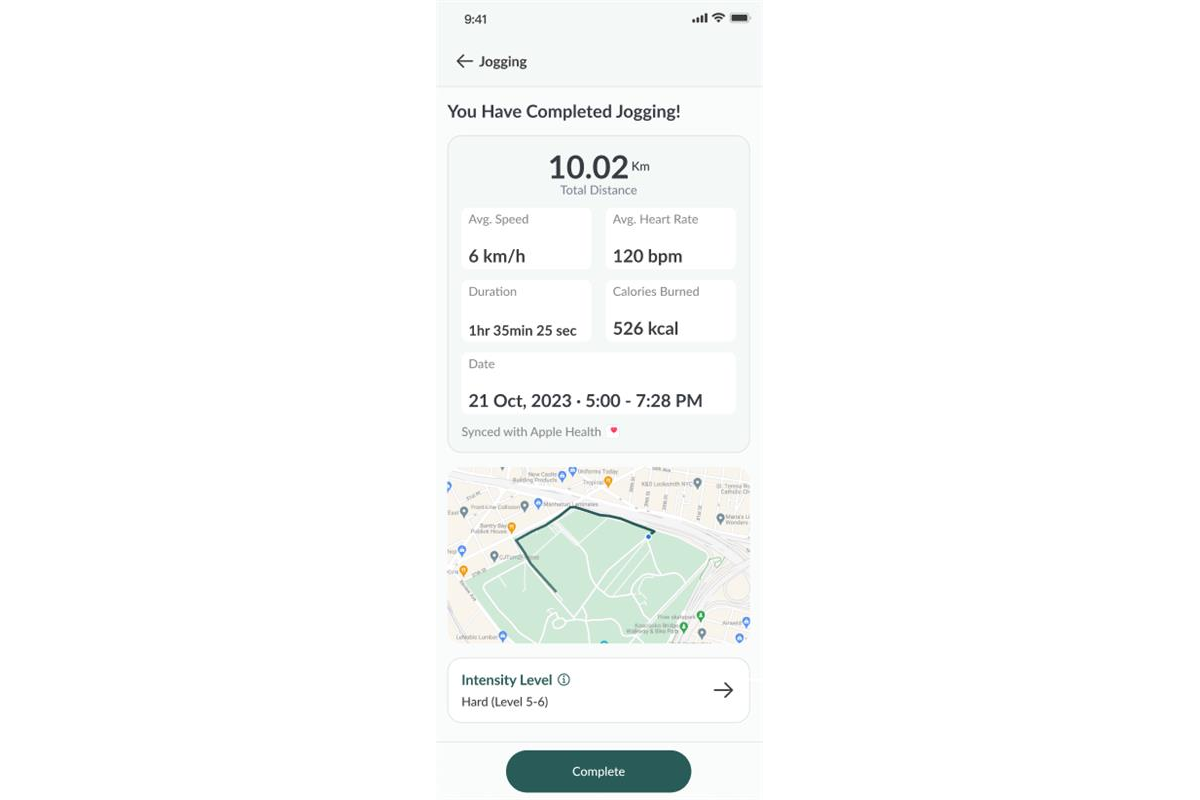
Exercise logging feature.

A weekly exercise plan is created based on each participant’s current level of physical activity and mobility to recommend the duration and intensity of exercise most suited for them. Participants can record the type, duration, and intensity of their exercise for the day by synchronizing data from their smart watch or through manual entry (Figures 3 and 4).

##### Diet

**Figure 5:**
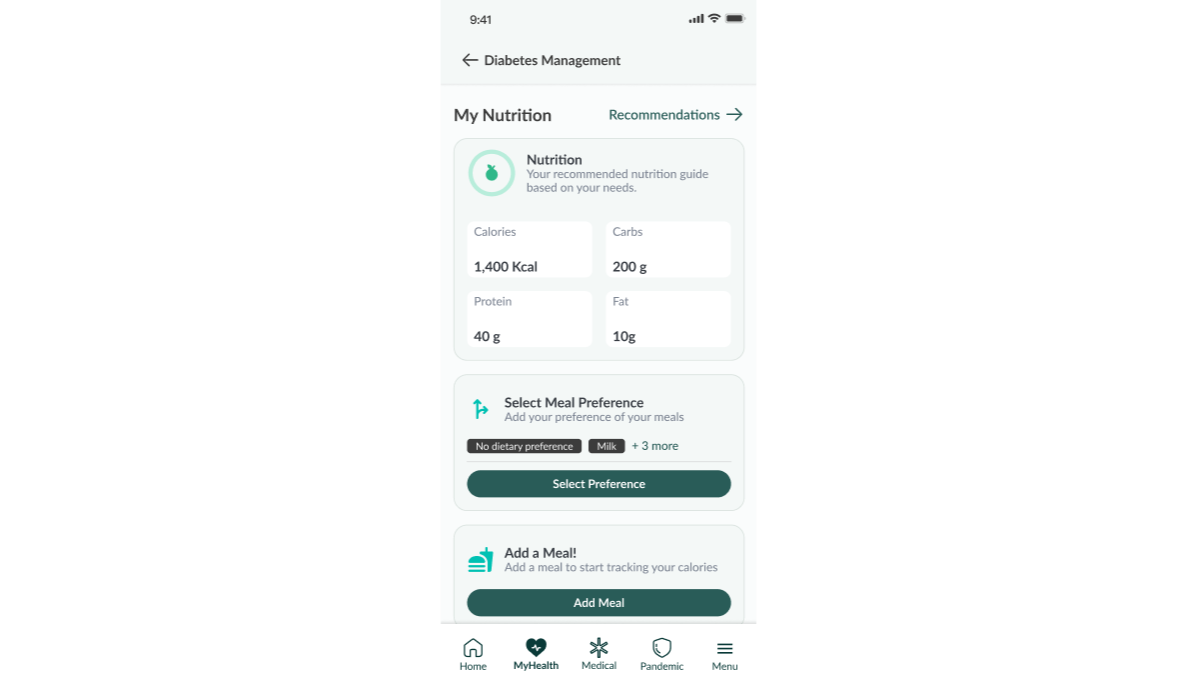
Meal plan.

**Figure 6:**
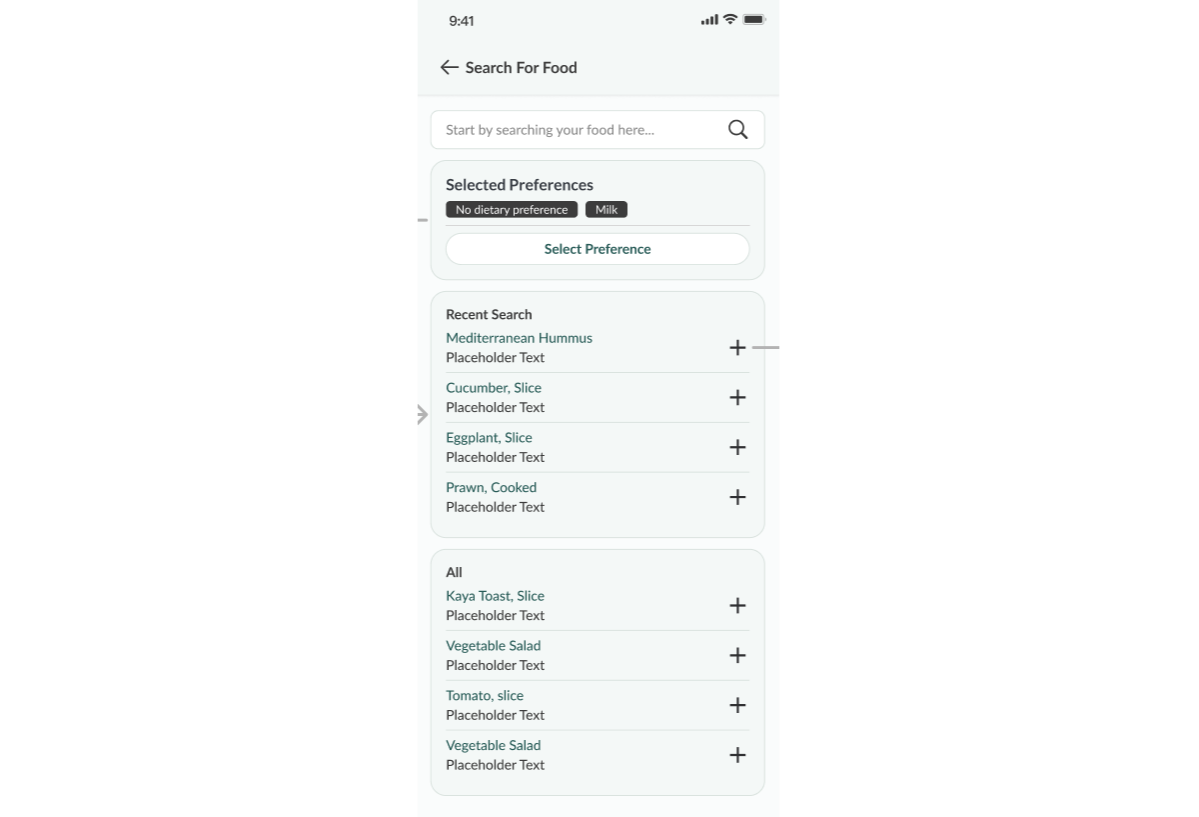
Food logging feature (food database).

**Figure 7:**
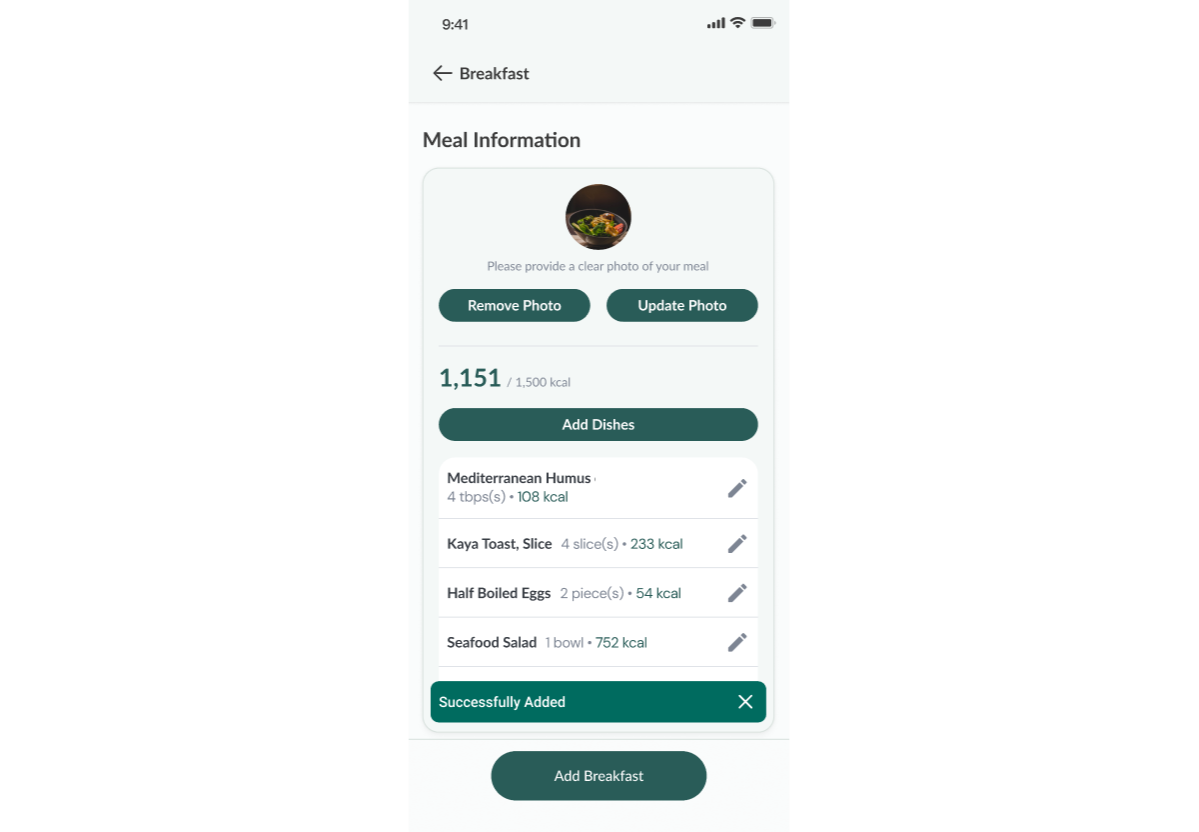
Food logging feature (meal information).

A daily nutritional plan, detailing recommended total calorie intake, including carbohydrate, fat, and protein intake across 3 meals (breakfast, lunch, and dinner), is created based on the participant’s demographic data and physical activity level. Participants have access to a localized food database to help log their meals (Figures 5-7).

##### Other Features

**Figure 8:**
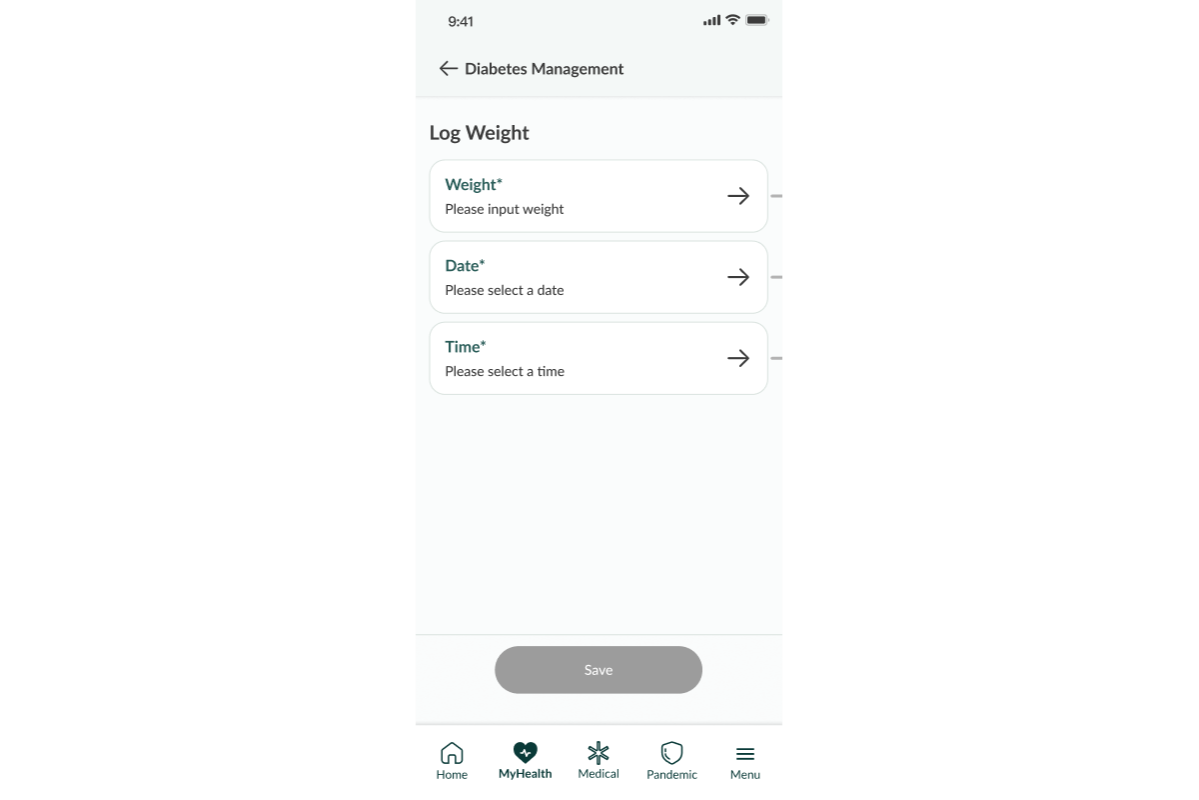
Weight logging feature.

**Figure 9:**
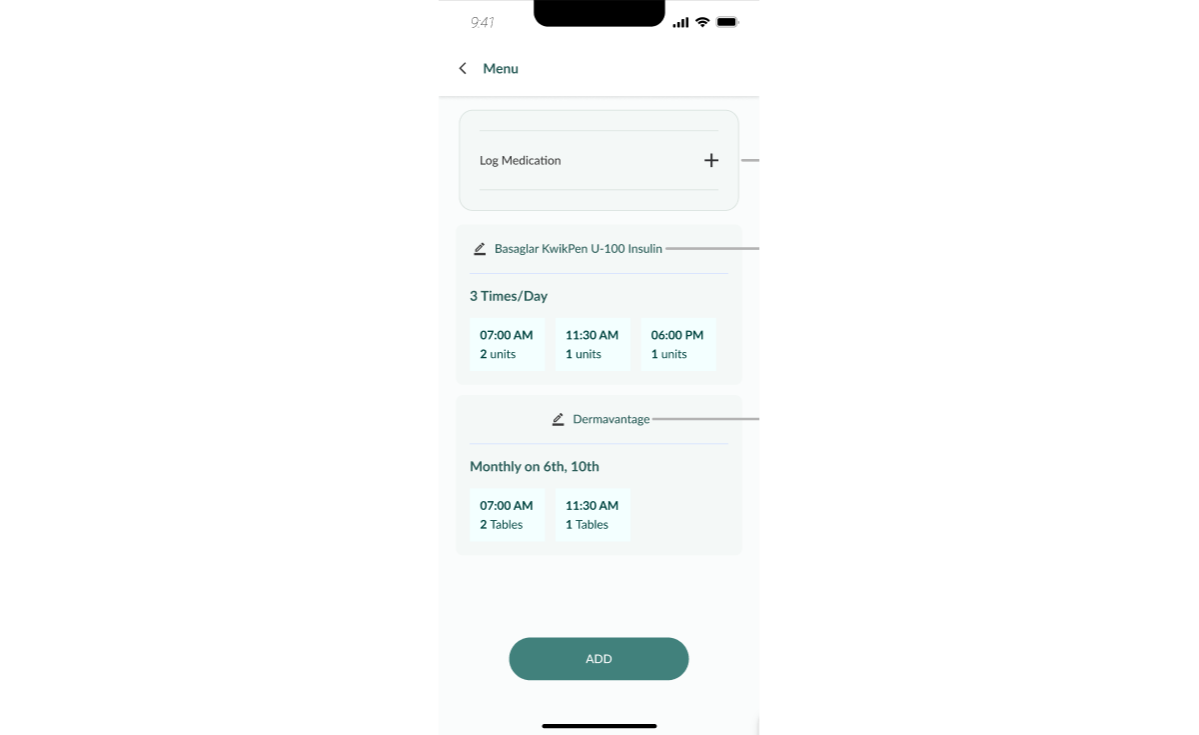
Medication log.

**Figure 10:**
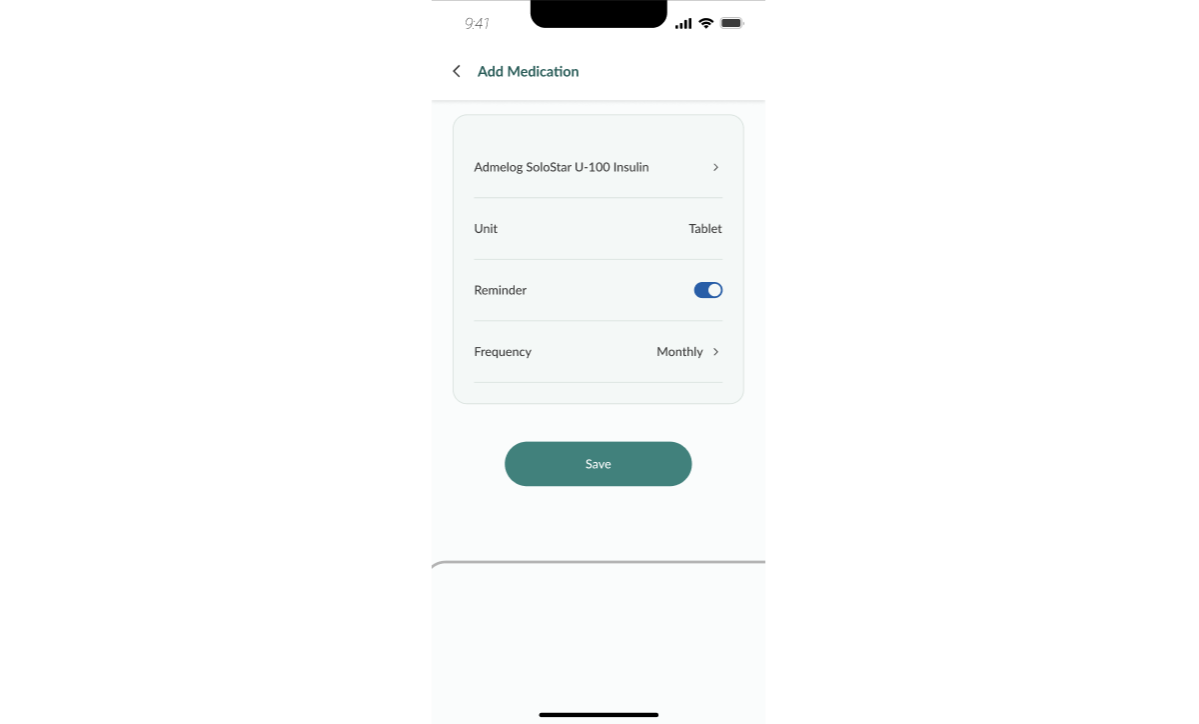
Reminders.

Other logging features, such as weight logging (Figure 8), sleep logging, and mood logging, are available for users to keep track of other parameters. Participants are also able to synchronize their medication list from the national electronic medical records, BruHIMS, into a medication logging function to remind them to take their medication in a timely manner (Figures 9 and 10).

Participants receive daily nudges to remind them to log their daily progress. They also receive reports that outline their blood glucose monitoring, and dietary and exercise habits to monitor their changes throughout the program.

#### Health Coach Support

##### Overview

Health coaches manage and communicate with their participants through the HCP portal. The portal, designed for health coaches and relevant HCPs, enables access to the treatment plan for close monitoring and further action.

Each participant’s assigned health coach conducts 5 scheduled video consultations with participants in the 16-week program. Health coaches review information logged by participants, track the changes in their anthropometric measures and habits, and provide additional guidance and advice to enable positive change in measured parameters. Sessions are aimed at reinforcing materials learned and emphasizing sustaining healthy habits for long-term management.

In addition to the regular video consultations, participants can engage with their health coaches through instant messaging. Health coaches also actively engage with participants to discuss their well-being, recent blood glucose readings, any symptoms, and adherence to prescribed medications. Health coaches also encourage participants to share details about their diet, exercise, and other factors that influence their blood glucose levels.

Additionally, health coaches respond to hypoglycemic events. During these times, if a hypoglycemic event alert is received, a health coach will initiate a video call to assess the situation based on the symptoms and signs presented by the participant.

##### Event Safety Monitoring

Health coaches are trained to identify and escalate cases of suspected diabetic emergencies, such as diabetic ketoacidosis, hyperosmolar hyperglycemic syndrome, and symptomatic hypoglycemia. Participants or their emergency contacts will be contacted to direct them to emergency services or to provide advice on blood glucose management based on the study protocol.

##### Escalation Model

The escalation pathway for managing participants in this study begins with a health coach assigned to the participant. Participants with hyperglycemia (defined per the study protocol) will be assigned to a dietitian or clinician in the research team for video consultation. Participants who experience (1) 2 or more episodes of hypoglycemia or (2) 3 or more episodes of hyperglycemia in a week despite dietetic interventions will be assigned to a clinical investigator for further evaluation.

#### Outcome

##### Primary Outcome

The primary outcome is to measure the proportion of participants with a reduction in HbA_1c_ of at least 0.6% at the end of the study.

##### Secondary Outcomes

The secondary outcomes include the following: (1) absolute change in HbA_1c_; (2) changes in BMI, waist circumference, fasting blood glucose, and fasting lipid panel (total cholesterol, low-density lipoprotein cholesterol, high-density lipoprotein cholesterol, and triglycerides) at the end of the study compared to baseline readings; (3) to describe the rate of fasting, rate of hypoglycemia, frequency of blood glucose monitoring, and fasting experience of Muslim participants in 2024; and (4) to calculate fasting risk using the IDF-DAR score

### Data Collection, Management, and Analysis

#### Data Collection

Data for the study is collected from different channels as follows:

1. BruHIMS for blood test results (baseline and final), eligibility data, and medication data
2. BruHealth app for lifestyle data through logging and wearable data synchronized with the app
3. Health coach portal for eligibility and monitoring data

#### Data Security

Participant data will be captured within the HCP portal software app developed in conjunction with the DM DTx app. Data access is in compliance with the Ministry of Health regulatory requirements. The analysis will be conducted on password-protected computers.

### Statistical Analysis

Descriptive statistics will be calculated to summarize the demographic profile of the research population and the primary and secondary outcomes outlined earlier. The mean, median, SD, and IQR will be reported for continuous variables, whereas frequencies and percentages will be used for categorical variables. Depending on the distribution of independent variables, appropriate paired statistical tests, such as the paired *t* test or the Wilcoxon signed-rank test, will be used to assess statistically significant differences between outcomes before and after treatment for both primary and secondary measures. Additionally, subgroup analyses will be conducted to assess for any significant differences in primary and secondary outcomes based on patient classifications, such as BMI categories, diabetic medication use profiles, or levels of engagement with the educational modules and health coaches in the BALANCE program.

### Withdrawal

Participants are withdrawn from the study if it is deemed that continued participation in the study poses significant health risks to them. These considerations include the following: (1) participants who require hospitalization during the study period (which may or may not be related to the study), (2) those deemed by clinical investigators to require immediate adjustments to their diabetes medications as delay may cause harm, and (3) those who fail to provide 2 local emergency contacts.

### Ethical Considerations

An online participant information sheet and consent form are available on the DM DTx module in the BruHealth app. Interested individuals are required to complete the online consent form before proceeding to complete the screening questionnaire. All participants are informed that their personal information will be kept confidential and securely stored within the Brunei Health Information Management System and BruHealth platforms, which follow national data protection and privacy standards. Identifying details will not be collected or published unless doing so is essential for scientific purposes, and no identifiable information (including names, initials, or images) will appear in any reports, publications, or supplementary materials. Participants have also been informed that they may withdraw from the study at any time without any effect on their access to routine health services. Ethics approval was granted by the Ministry of Health, Brunei Darussalam Medical and Health Research Ethics Committee (MHREC/MOH/2024/111).

## Results

**Figure 11:**
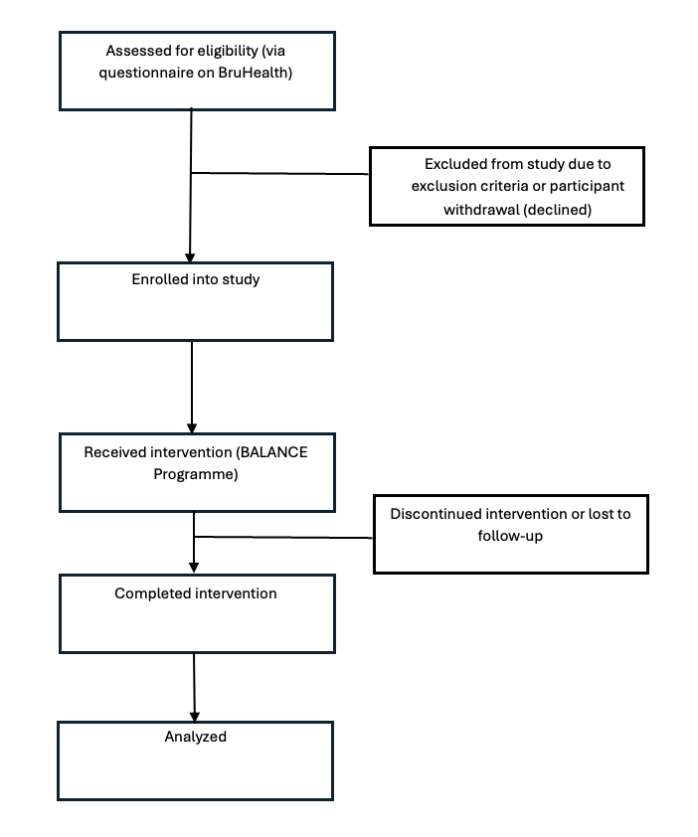
CONSORT (Consolidated Standards of Reporting Trials) flowchart: the recruitment process of the BALANCE program.

Recruitment began on August 20, 2024, following project approval in July 2024, and continued until July 2025. By the end of data collection on November 12, 2025, a total of 459 participants had been enrolled, and 422 (91.9%) had completed the program. Data analysis is currently ongoing, with results expected in early 2026 (Figure 11).

## Discussion

### Anticipated Findings

With the rising prevalence of diabetes in Southeast Asia, organizations such as the International Diabetes Federation are increasing efforts in diabetes advocacy and exploring new ways to improve access to quality health care and affordable treatments. Patient empowerment by incorporating self-management represents an important component of diabetes care, which will improve health outcomes and reduce diabetes-related complications. Therefore, new interventions should be designed with a focus on incorporating the latest clinical guidelines with an all-encompassing program that includes monitoring, diabetes education, maintaining a healthy lifestyle through diet and physical activity, medication tracking, and motivational coaching. A reduction of 1% in HbA_1c_ levels over 3 months is associated with decreased mortality and a reduction in microvascular disease, while a reduction in weight has been shown to improve glycemic control and insulin sensitivity, and potentially reduce medication dosage [21,22]. Consequently, an effective program would focus on methods to improve key cardiovascular risk factors, such as a reduction in HbA_1c_ and weight.

The increasing digitalization in the health care landscape has led to the deployment of mHealth and digital interventions to guide self-management of chronic conditions, particularly in diabetes management. Diabetes management apps can supplement traditional face-to-face care. This will help reduce the time and resources required from the health care system. Patients who have better self-management practices tend to manage their condition better [9]. By allowing users to actively participate in their own health management, users can increase their health literacy and become more confident in navigating their condition. However, an issue faced by most diabetes management apps is low engagement, with many users lost to follow-up [23]. A study showed that users were less likely to engage due to the high effort and difficulty in using the apps [24]. Therefore, collecting feedback on participant experience is crucial to identify functions and protocols that need to be redesigned to create an app that enhances the patient experience and level of engagement.

Due to the global burden of diabetes, various apps for diabetes management are commercially available on the market [25]. The main functionalities offered include self-management tasks (medication, blood glucose monitoring, exercise, and food intake), weight and blood pressure tracking, and education [26]. Most apps cover at least 1 self-management task, with blood glucose monitoring being the most common function [26]. According to the research that has been conducted to assess the efficacy of these apps, diabetes self-management apps have demonstrated a significant reduction in HbA_1c_ and body weight after a 6-month follow-up [9,27]. Most of these apps are free to download, with premium functions that require additional fees. The average cost of diabetes management apps on the market is between US $4.57 and US $5.03, with a higher annual cost for apps with multiple functions, making mHealth a cost-effective solution focused on preventing disease progression [28].

DM DTx is the first digital intervention solution of its kind in Southeast Asia. Most diabetes mHealth solutions are targeted at the North American market as part of an annual subscription plan usually provided by employers. This is largely attributed to the increasing penetration of smart consumer devices and the higher acceptance of mHealth solutions in the region [28]. Additional research on the efficacy of diabetes self-management apps in Southeast Asia should be conducted, as recommendations from solutions on the market may not be suitable in Southeast Asia due to different cutoff ranges for clinical parameters and cultural differences. Lifestyle habits and user behavior may also differ vastly between different populations, solidifying the need for region-specific intervention designs. Most commercially available diabetes management apps showed only low-to-moderate success in terms of user adherence, proving that diabetes self-management solutions do not follow a one-size-fits-all approach.

This study possesses some limitations. First, the study is designed based on volunteer sampling. Ideally, the study should be designed as a randomized controlled trial to reduce selection bias and ensure that the results are generalizable to a broader population. Any observable effects cannot be conclusively attributed to the intervention due to the lack of a control group. Sampling is done within the Bruneian population, so the results may not apply to populations outside of the current demographic. As the participants are not followed long-term, there is a lack of understanding of the sustained effects and durability of the intervention over time. Participants’ readiness to change their lifestyle habits was also not measured, as it was assumed that their participation translated to readiness. Finally, participants’ digital literacy is not measured during recruitment, which may affect their participation in the digital intervention and lead to less optimal results due to external barriers.

### Conclusions

Self-management mHealth apps remain a promising adjunct to chronic disease care, especially in T2DM. However, the impact of these apps in improving health is greatly dependent on localization to a region’s clinical and cultural context. Through data-driven research, gaps in intervention and app design can be identified to bridge the gap between theorized and realized improvements in health outcomes.

Ultimately, this research aims to understand the efficacy of DM DTx to develop a solution that is fit for purpose, clinically sound, patient-centered, and sufficiently personalized.

## Abbreviations

BruHIMS: Brunei Darussalam Health Care Information and Management System
DM DTx: diabetes mellitus digital therapeutics
DTx: digital therapeutics
HbA_1c_: glycated hemoglobin
HCP: health care professional
IDF-DAR: International Diabetes Federation-Diabetes and Ramadan Alliance Risk
mHealth: mobile health
SMART: specific, measurable, achievable, relevant, and time-bound
T2DM: type 2 diabetes mellitus
